# Utilization of a recombinant defensin from Maize (*Zea mays* L.) as a potential antimicrobial peptide

**DOI:** 10.1186/s13568-020-01146-9

**Published:** 2020-11-25

**Authors:** Najla Amin T. Al Kashgry, Hussein H. Abulreesh, Iman A. El-Sheikh, Yaser A. Almaroai, Reda Salem, Ismail Mohamed, Fatma R. Waly, Gamal Osman, Mahmoud S. M. Mohamed

**Affiliations:** 1 Biology Department, College of Science, Taif University, Taif, Saudi Arabia; 2 Department of Biology, Faculty of Applied Science, Umm Al-Qura University, Makkah, Saudi Arabia; 3 Research Laboratories Center, Faculty of Applied Science, Umm Al-Qura University, Makkah, Saudi Arabia; 4 Agricultural Genetic Engineering Research Institute (AGERI), ARC, Giza, 12619 Egypt; 5 Department of Botany and Microbiology, Faculty of Science, Cairo University, Giza, 12613 Egypt

**Keywords:** *Zea mays l.*, Antimicrobial, Defensin, Recombinant, Heterologous expression

## Abstract

The search for effective and bioactive antimicrobial molecules to  encounter the medical need for new antibiotics is an encouraging area of research. Plant defensins are small cationic, cysteine-rich peptides with a stabilized tertiary structure by disulfide-bridges and characterized by a wide range of biological functions. The heterologous expression of Egyptian maize defensin (MzDef) in *Escherichia coli* and subsequent purification by glutathione affinity chromatography yielded 2 mg/L of recombinant defensin peptide. The glutathione-S-transferase (GST)-tagged MzDef of approximately 30 kDa in size (26 KDa GST +  ~ 4 KDa MzDef peptide) was immunodetected with anti-GST antibodies. The GST-tag was successfully cleaved from the MzDef peptide by thrombin, and the removal was validated by the Tris-Tricine gel electrophoresis. The MzDef induced strong growth inhibition of *Rhizoctonia solani*, *Fusarium verticillioides*, and *Aspergillus niger* by 94.23%, 93.34%, and 86.25%, respectively, whereas relatively weak growth inhibitory activity of 35.42% against *Fusarium solani* was recorded. Moreover, strong antibacterial activities were demonstrated against *E*. *coli* and *Bacillus cereus* and the moderate activities against *Salmonella enterica* and *Staphylococcus aureus* at all tested concentrations (0.1, 0.2, 0.4, 0.8, 1.6, and 3.2 µM)*.* Furthermore, the in vitro MTT assay exhibited promising anticancer activity against all tested cell lines (hepatocellular carcinoma, mammary gland breast cancer, and colorectal carcinoma colon cancer) with IC_50_ values ranging from 14.85 to 29.85 µg/mL. These results suggest that the recombinant peptide MzDef may serve as a potential alternative antimicrobial and anticancer agent to be used in medicinal application.

## Introduction

The higher plants are experiencing a diverse array of biotic stresses such as insects, herbivores as well as diseases caused by phytopathogenic bacteria and fungi throughout their lives in the natural environment. These different factors decrease plant productivity due to physical damages and physiological and molecular changes they cause on the growth and development of plants (Singh et al. 2020). To counter the invasions of phytopathogens, plants have an enormous variety of secondary metabolites involved in distinctive innate defense mechanisms, pathogenesis-related proteins in plant defense mechanisms, and plant defensins (Dixon 2001; Abdelmohsen et al. 2020; Mostafa et al. 2009; Parisi et al. 2019; Yehia et al. 2020).

The α-defensins, one group of plant defensins, are small cationic peptides with a molecular weight of approximately 5 kDa, which belong to the family of antimicrobial peptides (AMP) and adopt an amphipathic structure with a wide range of biological functions such as antibacterial, antifungal, antiviral, and anticancer activity without toxicity to mammalian cells (van der Weerden and Anderson 2013). In general, the structure of defensins is characterized by conserved cysteine-rich peptides with the stabilized tertiary structure of the folded α-helix and β-sheet compacted by three to four intramolecular disulfide bonds (Lacerda et al. 2014). The defensin molecules are chemically stable because their compacted structure provides the advantage of resisting extreme temperatures and protease degradation (Rodríguez-Decuadro et al. 2019).

The similar structure of defensins was conserved in invertebrate and vertebrate β-defensins. The β-defensins are also involved in the defense system of vertebrates, exhibit a wide range of antimicrobial activities and share similar structures, α-helix, and β-sheet, but with a different topology compared to α-defensins (Montero-Alejo et al. 2012; Shafee et al. 2016).

Protein sequence analysis of different plant defensins revealed the significant amino acid variability except for the cysteine peptides and few other residues, but conservation of their three-dimensional structures was noticeable (Lacerda et al. 2014; Parisi et al. 2019).

Gene expression analysis of plant defensins indicated that genes are either constitutively expressed or up-regulated in response to biotic/abiotic stresses in all plant tissues or specific plant organs (Noonan et al. 2017; Parisi et al. 2019). Recently, high-level expression of different defensin recombinant proteins has been reported. Hence, large-scale production is feasible (Guillén-Chable et al. 2017; Rodríguez-Decuadro et al. 2019).

Although many sequences of plant defensins are available, relatively few ones have been characterized to date. Therefore, in this study, MzDef, a maize defensin peptide, was heterogeneously expressed, and in vitro antimicrobial activities against phytopathogenic microorganisms as well as anticancer activities were evaluated.

## Materials and methods

### Plant seeds, bacterial and fungal strains

The seeds of Egyptian maize hybrid cultivar Gz 168 were kindly provided by the Maize Department, Field Crops Research Institute, ARC, Giza, Egypt. The reference fungal strains used in this study were obtained from Mycological center, Assuit University, Egypt; *Fusarium solani* AUMC 10391, *Fusarium verticillioides* AUMC 2652.1, *Rhizoctonia solani* AUMC 6594, and *A. niger* AUMC 4301. The bacterial strains were obtained from Microbiological Resources Center (Cairo Egypt); *Bacillus cereus* EMCC 1006, *Staphylococcus aureus* EMCC 1351, *Salmonella enterica* EMCC 1038 and *Escherichia coli* ATCC 25922 from the American Type Culture Collection (ATCC).

### Isolation, cloning, and expression of the defensin coding sequence from maize

Total genomic DNA was isolated from leaf tissues (10–20 mg) of Gz 168 hybrid cultivar of Egyptian maize (*Zea mays L.*) by CTAB method (Guzmán et al. 2020). A pair of degenerate primers, forward: 5′-ACTAGCAKAYCTTCTTGCAGA-'3, and reverse: 5′-GATGGCKCYGTCTCGWCG-'3, was designed, according to the putative defensin sequence of the maize available in the GenBank database using the Lasergene software, MegAlign version 4.0 and primer select version 4.0.

*Pfu* DNA polymerase-generated PCR products were cloned into the pJET1.2/blunt cloning vector (Thermo Fisher Scientific, CA, USA) according to the manufacturer’s instructions. The sequence (GenBank Acc. No.: MT621394) of defensin gene isolated from Egyptian maize was previously determined and characterized (Amin et al. 2021). Upon the obtaining sequence, a pair of specific primer was designed (sense: 5′- CGC*GGATCC*AGCAGCAGCAACTGCGCC-3′, and the anti-sense: 5′- CCG*CTCGAG*CTAGCAGTTCTTGCAGAAGC-3′ flanked by *Bam*HI and *Xho*I recognition sites (*italic*), respectively) for subcloning into pGEX-4 T-1 prokaryotic expression vector. The defensin coding sequence was inserted in-frame to be fused with GST-tagged protein to facilitate the further purification (Salem et al. 2019a; El-Gaied et al. 2020).

### Heterologous expression of the MzDef protein

The pGEX-4 T-1 harboring recombinant defensin (pGEX-4 T-1-Def) was transformed into the BL21 (DE3) *E. coli* strain (Elgaied et al. 2017; Elmenofy et al. 2020a). Positive colonies were selected on the basis of their growth on Luria–Bertani (LB) agar plates supplemented with 50 µg/mL ampicillin. The 5 mL LB medium containing ampicillin was inoculated with a single isolated colony and grown overnight at 37 °C. The expression of the GST-defensin fusion was induced by the addition of 0.1 mM IPTG. The bacterial pellet was collected by centrifugation, and the recombinant protein was batch purified with Glutathione Sepharose 4B resin (Sigma, St Louis, USA). In an overhead shaker, the filtered bacterial lysate was incubated with 2 mL of glutathione Sepharose and left overnight at 4 °C. The unbound proteins were washed twice with 10 mL of GST binding buffer, followed by two washes with 10 mL of GST binding buffer containing 1% Triton X-100 to remove nonspecifically bound proteins. The bound recombinant GST-defensin peptide was eluted with 1 mL of elution buffer (50 mM Tris–HCl pH 8.0, 400 mM NaCl, and 10 mM reduced glutathione). The N-terminal GST-tag was cleaved by overnight digestion of thrombin, and then the purity of the recombinant protein was analyzed by Tris-Tricine gel electrophoresis, and its concentration was estimated by Bradford assay.

### Western blot analysis

To confirm the purification of the defensin-GST fusion protein and to determine the cleavage of the GST-tag from defensin, 2 µg of the purified protein was separated in a 15% (w/v) Tris-Tricine gel. After electrophoresis, proteins were stained with Coomassie R–250 in 10% ethanol (v/v) and 50% acetic acid (v/v), followed by destaining with 12.5% (v/v) isopropanol and 12% (v/v) acetic acid. The separated proteins were electro-transferred to a polyvinylidene difluoride membrane (PVDF, Thermo Scientific, US). The antibodies against GST protein were used as a primary antibody. Anti-mouse-Alkaline Phosphatase Sigma-Aldrich (St. Louis, MO) was used as a secondary antibody. Nitro blue tetrazolium chloride (NBT) and 5-bromo-4-chloro-3′-indolyphosphate p-toluidine (BCIP) were used as substrates for detection (Salem et al. 2018; Salem et al. 2019b; Elmenofy et al. 2020b).

## Evaluation of the antimicrobial activity of the peptide MZ-Def

### Quantitative antifungal activity

The growth rate of fungi is presented as the percentage of fungal growth inhibition. Briefly, tests were performed in 96-well microtiter plates, each well containing 100 µL of potato dextrose broth (PDB) medium, fungal spore densities of 3 × 10^6^ spores/mL suspension in saline with 0.05% Tween 20, and different concentrations (0.1, 0.2, 0.4, 0.8, 1.6, and 3.2 µM) of the purified peptide MZ-Def with three replicates. Wells containing no peptide served as controls. The plates were incubated in the dark at 35 °C for three days. At 24-h intervals, absorption (A) readings at 595 nm were recorded and corrected by subtracting time zero readings from the sample readings. The percentage growth inhibition is defined as the ratio of the corrected A_595_ of the control minus the corrected A_595_ of the sample over the corrected A_595_ of the control multiplied by 100 (CLSI 2012).

### Evaluation of antibacterial activity using the microbial growth curve

The microbial growth curve was used to test the antibacterial activity of the protein MZ-Def against different bacterial species (*E. coli* and *S. enterica* as Gram-negative and *S. aureus*, and *B. cereus* as Gram-positive). Briefly, the bacterial cells were cultured overnight, and on the following day, 100 µL of freshly grown bacterial culture was added to 5 mL of LB medium. When the read at 600 nm reached 0.6, 100 µL of purified MZ-Def solution (concentration gradients of 0.1 to 3.2 µM) was added to 5 mL of LB growth medium (with three replicates). Finally, the read values at 600 nm were determined for each bacterium. Approximately 3 mL of LB growth medium without the purified protein MZ-Def was regarded as the negative control (with three replicates). The antibacterial activity against a given bacterial species was assessed by determining the read values at 600 nm after 24 h of incubation.

### Determination of the antimicrobial activity by agar well diffusion method

Agar well diffusion assay was performed according to the bacterial protocol MO2-A12 described in the instructions by the Clinical and Laboratory Standards Institute (CLSI) (CLSI, 2015). Briefly, inoculum containing 10^6^ CFU/mL of each microbial culture was spread on nutrient LB agar plates, selected for the bacteria, with a sterile swab, as well as potato dextrose agar (PDA) plates with 5 mm disc, selected for the fungi. Subsequently, wells of 8 mm diameter were punched into the agar medium, filled with 100 µL of purified MZ-Def protein in PBS (pH 7.4) with final concentrations of 0.1, 0.2, 0.4, 0.8, 1.6, and 3.2 µM (three replicates for MzDef against each organism), and allowed to diffuse at room temperature for 2 h. Wells containing PBS served as a negative control. The plates were then incubated in an upright position at 37 °C for 24 h (for bacteria) and at 28 °C for 48 h (for fungi). PBS-containing wells acted as a negative control.

### In vitro cytotoxicity

Hepatocellular carcinoma (HePG2), mammary gland breast cancer (MCF-7), and colorectal carcinoma colon cancer (HCT-116) cell lines were obtained from ATCC and holding company for biological products and vaccines (VACSERA), Cairo, Egypt. The cell lines were used to determine the inhibitory effects of the MZ-Def peptide on cell growth using MTT assay. This colorimetric test is based on the conversion of the yellow tetrazolium bromide (MTT) to a purple formazan derivative in the mitochondria of viable cells by succinate dehydrogenase. Cell lines were grown in RPMI-1640 medium (Sigma co., St. Louis, USA) supplemented with 10% fetal bovine serum (GIBCO, UK). The antibiotics (100 units/mL penicillin and 100 µg/mL streptomycin) were incubated in a 5% CO_2_ incubator at 37 °C. The cell lines were seeded into the wells of a 96-well plate at a density of 1.0 × 10^4^ cells per well at 37 °C, 5% CO_2_ for 48 h. The cells were treated with different MZDef concentrations and incubated for 24 h; thereafter, 20 µL of 5 mg/mL MTT solution was added to each well and incubated again for 4 h. In each well, 100 µL of dimethyl sulfoxide was added to dissolve the purple formazan. The quantity of formazan was measured by recording changes in absorbance (A) at 570 nm using a plate reader (Biochrom EZ 800, USA). The relative percentage of cell viability was calculated as follows: 100 X (A_570_ of treated samples/A_570_ of the untreated sample).

## Results

### Cloning, heterologous expression, and purification of Mz-Def

**Fig. 1:**
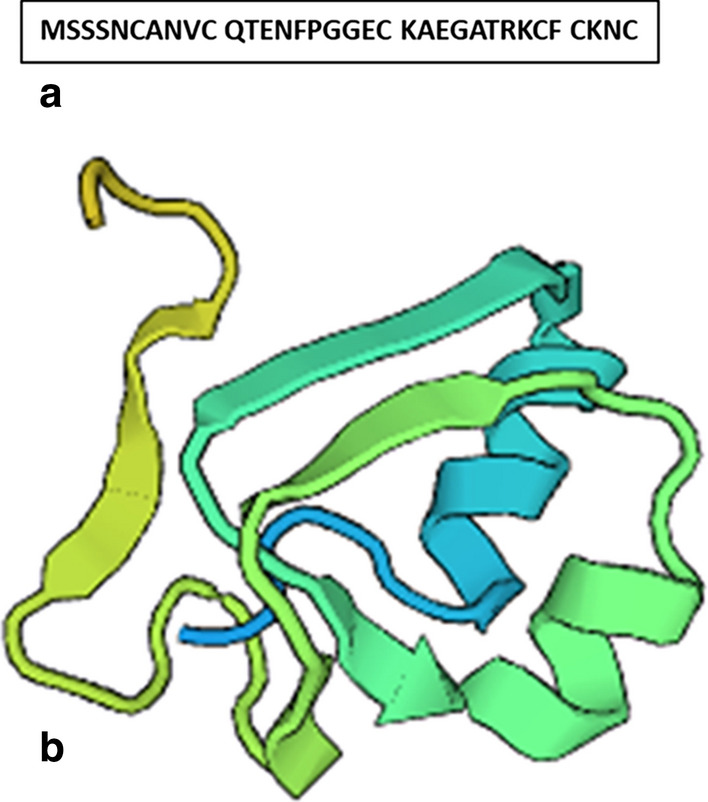
Analysis of defensin peptide coding sequence isolated from Egyptian maize (*Zea mays L.*). The isolated sequence consisted of a single open reading frame (ORF) of 108 bp encoding for predicted signal peptide of 34 amino acids long (**a**). The tertiary structure of its deduced amino acids generated by SWISS-Model homology analysis showed a common defensin tertiary structure of two α-helix and three antiparallel β-sheets, arranged as αβαββ and stabilized by the intermolecular disulfide bonds between cysteine residues (**b**)

In this study, a defensin coding sequence was PCR amplified from total genomic DNA isolated from Gz 168 hybrid cultivar of maize (*Zea mays L.*). The DNA fragment was successfully amplified with the expected molecular size of approximately 245 bp and then cloned into the pJET1.2/blunt cloning vector. The nucleotide sequence of MzDef gene was given GenBank Acc. No.: MT621394. Sequence analysis showed that the isolated sequence consisted of a single open reading frame (ORF) of 108 bp encoding the predicted signal peptide of 34 amino acids long (Fig. 1a). Homology analysis of the deduced amino acid sequence indicated that MzDef have a common defensin tertiary structure of two α-helix and three antiparallel β-sheets, arranged as αβαββ (Fig. 1b) and stabilized by the intermolecular disulfide bonds between cysteine residues.

**Fig. 2:**
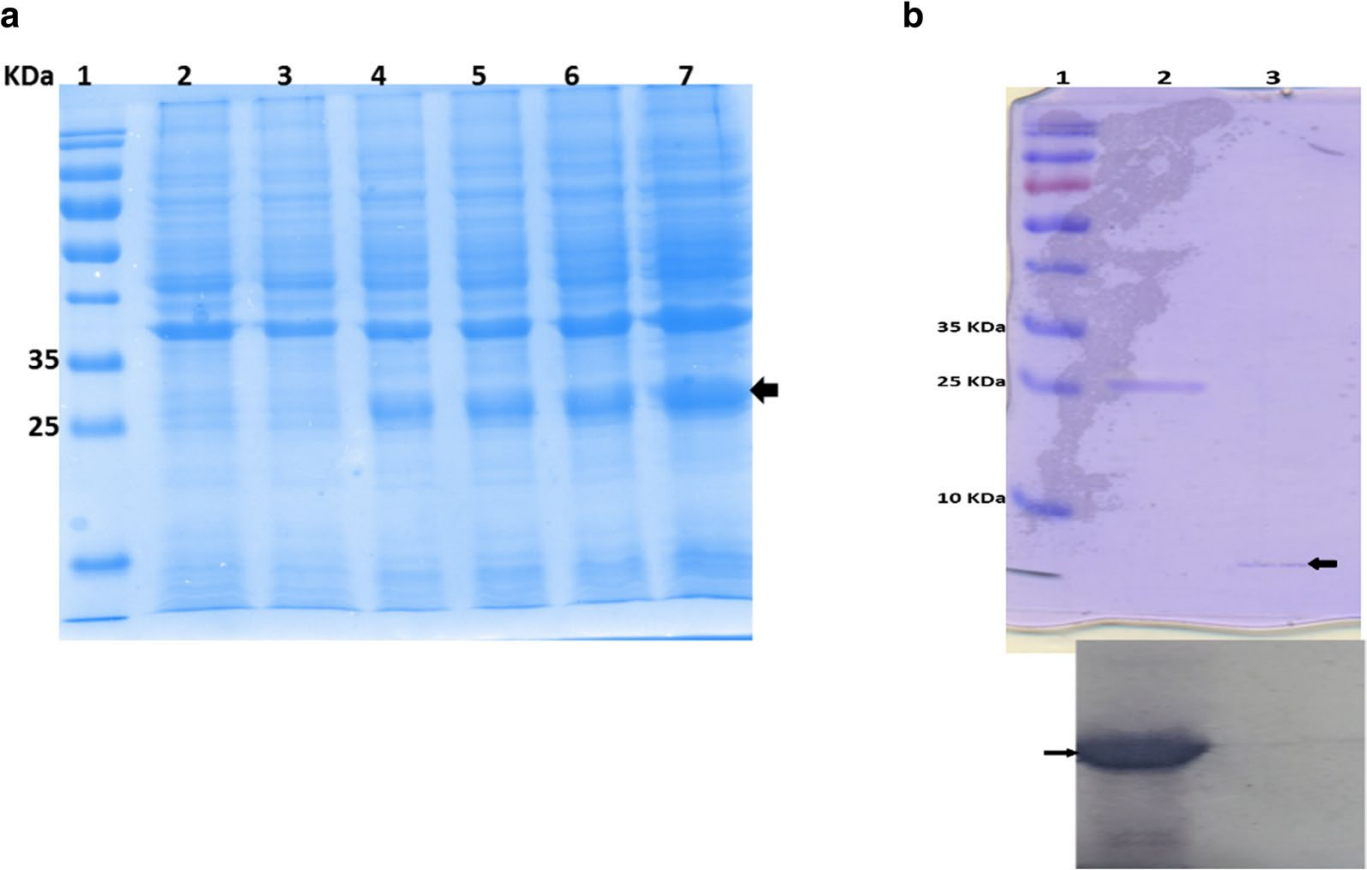
SDS-PAGE followed by Coomassie blue R-250 gel staining showing**: a** The induction time course for MzDef in *E. coli* BL21 plysS (DE3) + RIL, 1: marker, 2, 3, 4, 5, 6 and 7 are the total proteins extracted from Bl21 *E. coli* where, 2 is negative, 3 is before induction, from 4–7 are 2 h, 3 h, and 4 h post induction, respectively. **b** Purification of GST-tagged MzDef from *E. coli* BL21 (DE3). Coomassie stained SDS-PAGE gel showing purification of GST- MzDef. The fusion proteins are indicated by a solid arrow. The corresponding western blot analysis is shown (below) using anti-GST antibody. 1, Molecular weight marker indicated in kDa; 2, GST- MzDef fusion protein; 3, purified MzDef recombinant protein

The recombinant MzDef fused to the GST-tag was successfully produced in *E. coli* from the BL21 (DE3) pLysS + RIL expression system. Total proteins were extracted from the induced *E. coli* BL21 after its transformation with the recombinant pGEX-4 T-1-MzDef. The MzDef protein band fused with the GST protein was clearly observed in the time-course analysis after separating the total extracted proteins by SDS-PAGE and staining with Coomassie Brilliant Blue (Fig. 2a). The optimal expression time was 3 h after induction that led to produce a significant amount of fusion protein, and longer induction time did not result in any substantial increase in yield (Fig. 2a). Purification of the recombinant peptide by glutathione affinity chromatography yielded 2 mg/L of the purified Mz-Def peptide emerged as a clear band of approximately 30 kDa (26 KDa GST +  ~ 4 KDa MzDef peptide). The GST-tag was successfully cleaved from the MzDef peptide by thrombin, and the purification was validated by Tris-Tricine gel electrophoresis.

### Immunodetection of the recombinant MzDef fusion protein

In order to confirm the presence of the corresponding expressed protein, western blot was performed. In addition to the MzDef peptide after cleavage by thrombin, purified fusion proteins were resolved by SDS-PAGE on 12% gel (Fig. 2b). Separated proteins were electroblotted onto PVDF. Antibodies against GST protein were used as a primary antibody, and the universal anti-mouse conjugated to alkaline phosphatase was used as a secondary antibody. The detection method was conducted using NBT and 5-bromo-4-chloro-3′-indolyphosphate *p*-toluidine salt (BCIP) as substrates. Western blot analysis showed that the specific signal for the expressed recombinant GST-MzDef protein can be identified by the anti-GST antibody but not with the cleaved MzDef peptide (Fig. 2b).

### Antifungal activity of the recombinant MzDef peptide

**Fig. 3:**
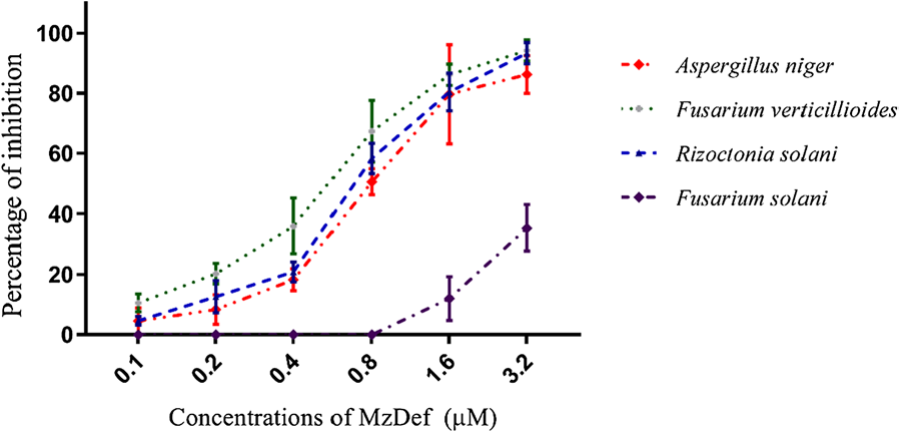
Antifungal activity of MzDef on the growth of *F. solani*, *F. verticillioides*, *R. solani*, *and A. niger*. The data is represented as a percentage of fungal growth as compared to the control reactions with no MzDef peptide. The standard error for each reaction was determined

**Fig. 4:**
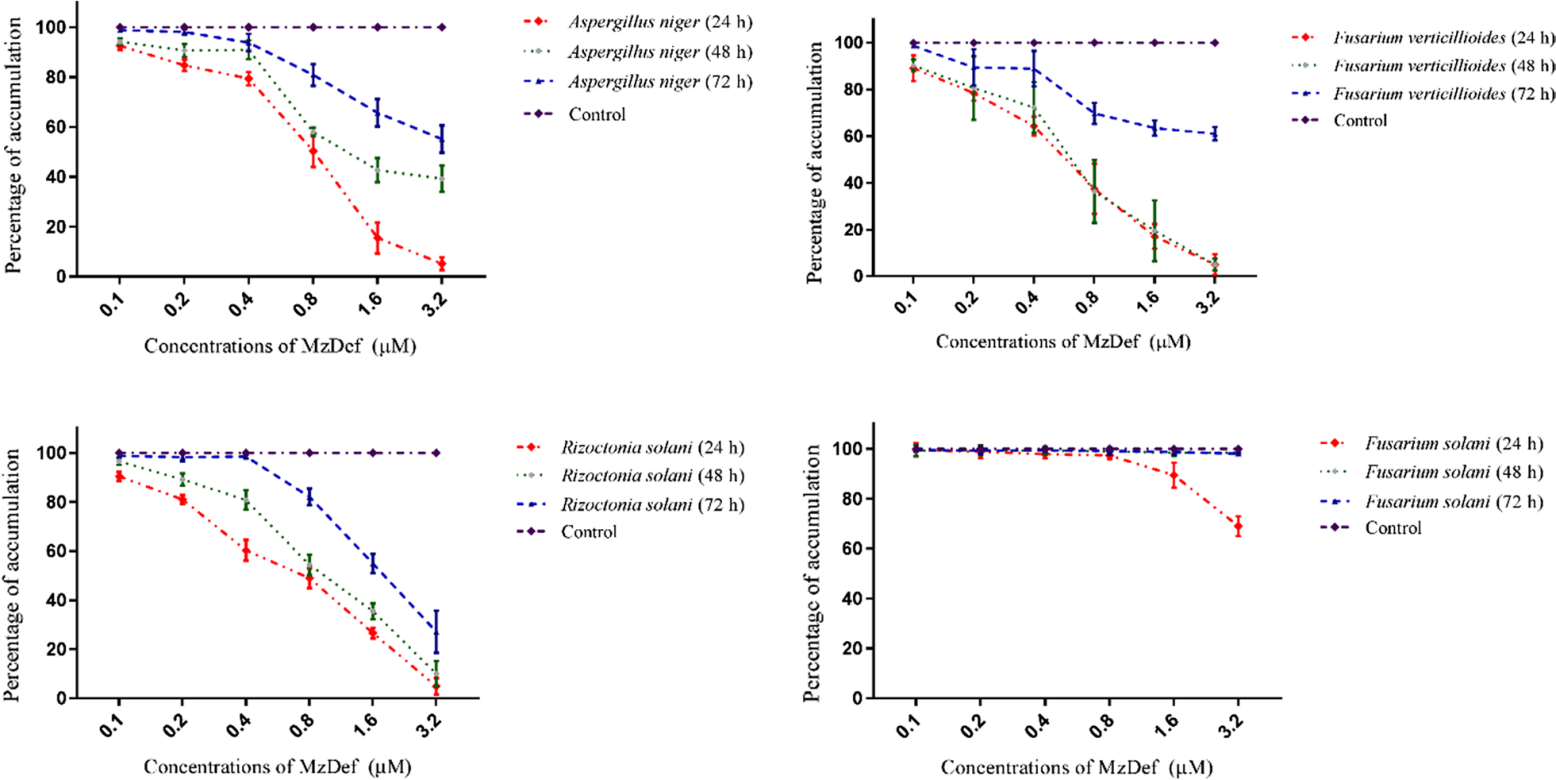
The effect of MZ-Def on the accumulation of *F. solani*, *F. verticillioides*, *R. solani*, and *A. niger* at different time interval 24, 48 to 72 h. The data is represented as a percentage of fungal accumulation as compared to the control reactions without MzDef peptide. The standard error for each reaction was determined

The antifungal activity of the recombinant MzDef was tested against several pathogenic fungi including *F. solani*, *F. verticillioides*, *R. solani*, and *A. niger* using a dose–response growth inhibition assay. The antifungal activity of MzDef was assessed by incubating the fungal spores in the presence of various concentrations (0.1, 0.2, 0.4, 0.8, 1.6, and 3.2 µM) of MzDef. The variation among technical replicates was less than 10%. At the highest concentration (3.2 µM) of MzDef, substantial inhibition of growth of the fungi *R. solani*, *F. verticillioides*, *F. solani*, and *A. niger* was observed. MzDef induced strong growth inhibition of the fungi *R. solani*, *F. verticillioides*, and *A. niger* by 94.23%, 93.34%, and 86.25%, respectively, whereas a relatively weak growth inhibitory activity (35.42%) against *F. solani* was recorded (Fig. 3). Furthermore, the effect of MzDef on the biomass accumulation of the fungi *F. solani*, *F. verticillioides*, *R. solani*, and *A. niger* was also determined over time (at 24, 48, and 72 h after incubation) (Fig. 4). The results revealed that the biomass accumulation of all tested fungi decreased with increasing the MzDef concentration. Nevertheless, with increasing the incubation time, the inhibitory effect became less effective. The highest concentration of MzDef (3.2 µM) decreased the accumulation of *A. niger* by 5.17%, and at 48 h and 72 h after incubation, it decreased by 39.62% and 55.17%, respectively (Fig. 4). The same level of accumulation (5.07%) was observed after 24 h and 48 h for *F. verticillioides* at the highest concentration (3.2 µM) of MzDef, while a higher level of accumulation (61.03%) was recorded after 72 h (Fig. 4). In contrast, a weak inhibitory effect against *F. solani* appeared when the accumulation decreased by only 68.67% after 24 h and disappeared afterward, and no effect was observed after 48 h and 72 h of incubation (Fig. 4). MZ-Def decreased the accumulation of *R. solani* by 4.94, 10.01, and 27.15% after 24, 48, and 72 h of incubation, respectively (Fig. 4).

These results were confirmed by the result of antifungal activity testing of the recombinant MzDef protein against the same species of fungi using agar well diffusion method, in which different concentrations (0.1, 0.2, 0.4, 0.8, 1.6, and 3.2 µM) of the recombinant MzDef protein were applied, and the relative antifungal activity with different inhibitory effects was observed for different fungal species (data not shown).

### Antibacterial activity of the recombinant MzDef

The antibacterial activity of the recombinant MzDef protein against different species of bacteria was evaluated. The determination of dose-dependent growth inhibition of bacteria was performed by measuring the absorption of the cultures at 595 nm in the presence of different concentrations of the recombinant MzDef. The results revealed that the inhibitory activity of the purified MzDef protein had different sensitivity for different bacterial species.

**Fig. 5:**
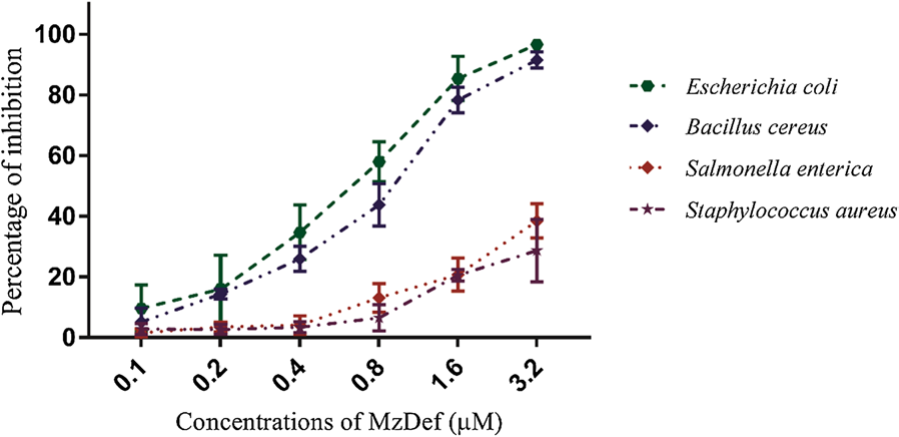
Growth inhibition of *E. coli*, *S. enterica*, *S. aureus*, *and B. cereus* determining the antimicrobial activity of MzDef. The data is represented as a percentage of bacterial growth inhibition as compared to the control reactions without MzDef peptide after 24 h of incubation. The standard error for each reaction was determined

The strongest inhibitory activity was observed against *E*. *coli* and *B. cereus* with the same effect, while the inhibitory effect against *S. enterica* and *S. aureus* was moderate. Furthermore, the antibacterial activity of the recombinant MzDef protein was tested against the same species of bacteria by applying different concentrations of the recombinant MzDef protein demonstrated that by increasing the concentration of the MzDef protein, the percentage of growth inhibition of bacteria was increased in all tested bacteria. At concentration 3.2 µM of the MzDef protein, the inhibition percentage recorded 96, 91, 39 and 29 against *E. coli*, *B. cereus S. enterica* and *S. aureus* respectively (Fig. 5).

### Evaluation of cytotoxicity of MzDef

**Fig. 6:**
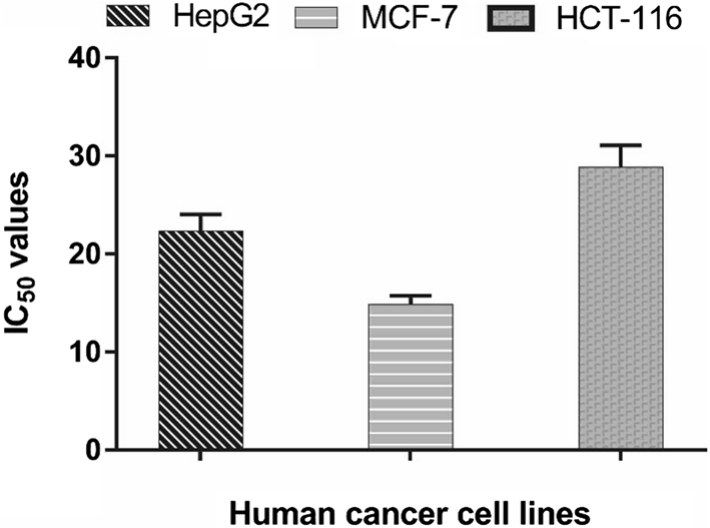
The in vitro cytotoxicity of MzDef as analyzed by MTT assay against human cancer cell lines: Hepatocellular carcinoma (HEPG-2), Mammary gland breast cancer (MCF-7) and Colorectal carcinoma Colon cancer (HCT-116). The bars on the graph represent mean ± SD of MzDef IC_50_ values of triplicate independent experiments (n = 3)

The MzDef peptide was screened for an in vitro anti-proliferative activity against three different cancer cell lines, including hepatocellular carcinoma (HEPG-2), mammary gland breast cancer (MCF-7), and colorectal carcinoma colon cancer (HCT-116). The tested MzDef peptide displayed promising anticancer activity against the cancer cell lines with IC_50_ values ranging from 14.85 to 29.85 µg/mL (Fig. 6).

## Discussion

Plants have an intrinsic defense system, and their innate immune systems are activated by the presence of external pathogens. This reaction is generally known as induced systemic resistance and provides a broad-spectrum resistance against a vast range of unassociated pathogens. During this process, a great number of resistance products like defensins are produced to prevent the spread and expansion of pathogens (Dowd and Johnson 2018; Lacerda et al. 2014; Hultmark et al. 1980; Cociancich et al. 1994; Lay and Anderson 2005; Boman et al. 1987; Kanost et al. 1990). Defensins are a category of cysteine-rich polypeptides with a low molecular weight of approximately 5 kDa and have antibacterial and antifungal activities (Guillén-Chable et al. 2017; Kant et al. 2009; Cornet et al. 1995; Garcia-Olmedo et al. 2015).

In the present study, firstly, a defensin coding sequence was isolated from Egyptian maize (*Zea mays* L.) and cloned. Subsequently, the MzDef peptide was obtained by the expression of cloned genes in *E. coli*. In vitro growth inhibitory activities of the recombinant MzDef against different species of bacteria (*E. coli*, *S. enterica*, *S. aureus*, and *B. cereus*) and fungi (*F. solani*, *F. verticillioides*, *R. solani*, and *A. niger*) were studied. The antifungal activities were confirmed by the increase in fungal biomass accumulation over time. These results are compatible with those previously reported by who found the inhibitory antifungal activity of ZmESR6, a defensin gene, isolated from other varieties of maize expressed in *E. coli*, against the bacterium *Rhizobium meliloti*, as well as the fungi *Fusarium oxysporum*, and *Plectosphaerella cucumerina*.

Furthermore, the expression of *Zea mays* defensin gene conferred transgenic tobacco plants enhanced tolerance to *Phytophthora* nicotianae var. *Parasitica*, and the recombinant protein displayed antifungal activity in vitro (Wang et al. 2011). A recent study reported that ZmDef124, a potential maize defensin-like protein, has inhibitory activity against both insects and fungi (Dowd and Johnson 2018). The fungal cell killing by defensins could be explained by several mechanisms such as disturbance of membrane permeability, the induced oxidative stress damage to DNA, and the induction of apoptosis and necrosis in cells (Aerts et al. 2011; Guilhelmelli et al. 2013).

In this study, MzDef was highly active against *R. solani*, *F. verticillioides*, and *A. niger* but was relatively effective against *F. solani*. The required concentration of plant defensins for inhibition of the growth of bacteria or fungi depends on the bacterial and fungal species and the plant defensing (Parisi et al. 2019; Wei et al. 2019).

In this study, MzDef demonstrated strong antibacterial activity against *E*. *coli* and *B. cereus* and moderate activity against *S. enterica* and *S. aureus* at all tested concentrations*.* The antibacterial activity is not a common feature of plant defensins; specific antibacterial activities against some species of bacteria have been reported (Parisi et al. 2019; Zhang and Lewis 2006). A recombinant mungbean defensin VrD1 was previously demonstrated to exhibit antibacterial activities against *Staphylococcus epidermidis* and *Salmonella typhimurium* (Chen et al. 2005). The substantial antimicrobial activities of defensins against bacteria and more than 40 different species of plant pathogens have been reported (Lay and Anderson 2005; Carvalho and Gomes 2009). For the defensins from maize, antibacterial activities against the Gram-positive plant pathogen *Clavibacter michiganensis* and the nitrogen-fixing bacterium *Rhizobium meliloti* were recorded (Balandin et al. 2005).

Moreover, eukaryotic defensins also exhibit anticancer activities (Lay and Anderson 2005, Guzmán-Rodríguez et al. 2016). In this study, the MzDef peptide was screened for an in vitro anti-proliferative activity against three different cancer cell lines, including hepatocellular carcinoma (HEPG-2), mammary gland breast cancer (MCF-7), and colorectal carcinoma colon cancer (HCT-116). The tested MzDef peptide displayed promising anticancer activity against the cancer cell lines with IC_50_ values ranging from 14.85 to 29.85 µg/mL.

The search for appropriate analogs as antibiotics has been the subject of several studies. Defensins have shown to be a useful alternative to antibiotics due to their unusual antibacterial function and the inability of some bacteria to develop antimicrobial resistance (Lay and Anderson 2005).

In this study, the full-length gene encoding MzDef, isolated from Gz 168 hybrid cultivar of local maize, was successfully cloned and expressed using prokaryotic systems. The MzDef peptide was purified to homogeneity, and the purity was confirmed by western blot analysis. The MzDef exhibited enhanced antibacterial and antifungal activities against different bacterial and fungal pathogens.

## Data Availability

All relevant data are within the manuscript.
